# The complete mitochondrial genome of *Abramites hypselonotus* (Günther, 1868) (Ostariophysi: Characiformes: Anostomidae): an aquarium headstander

**DOI:** 10.1080/23802359.2021.1930214

**Published:** 2021-05-23

**Authors:** Adma Kátia Lacerda-Chaves, Adriana Heloísa Pereira, Evanguedes Kalapothakis

**Affiliations:** a Biological and Health Sciences Center, Federal University of the West of Bahia, Barreiras, Brazil; b Department of Ecology, Genetics and Evolution, Laboratory of Biotechnology and Molecular Markers, Institute of Biological Sciences, Federal University of Minas Gerais, Belo Horizonte, Brazil

**Keywords:** Complete mtDNA, *Abramites hypselonotus*, aquarium fish, next-generation sequencing, marbled headstander

## Abstract

*Abramites hypselonotus*, commonly known as marbled headstander, is an important freshwater aquarium fish from Brazil, found in the Orinoco, Amazon, Paraguay and lower Parana River basins. This genus has only two species and only this species occurs in Brazil. The complete mitochondrial genome of *Abramites hypselonotus* is 16,685 bp in length and it includes 13 protein-coding genes (PCGs), 2 rRNAs, 22 tRNAs genes and a control region with 1,028 bp. It has two PCGs with GTG start codon and the others with ATG start codon. Four of the 13 PCGs appear TAA stop codon, three incomplete TA_ stop codon, four incomplete T_ _ stop codon, one contain AGG stop codon and one TAG stop codon. Phylogenetic analysis showed that *Abramites hypselonotus* formed a sister group of *Leporinus affinis* (AP011994.1), thus maintaining the Family Anostomidae as a clade.

*Abramites* Fowler (1906) is a basal genus of the Anostomidae family (Sidlauskas and Vari 2008) and it has only two species recognized (Garavello and Britski 2003; Albert et al. 2006): *Abramites hypselonotus* (Günther, 1868), cis-Andean specie and *Abramites eques* (Steindachner, 1878), trans-Andean specie (Vari and Williams 1987). *Abramites hypselonotus* is an important freshwater aquarium fish from Brazil, known as *headstanders*, because it has a different angle with its head down while resting (Gery 1977; Vari and Williams 1987). In Brazil, the capture, transport and trade of this species for ornamental purpose is authorized (Normative Instruction MPA/SAP n° 10, 17/04/2020).

However, ecological research shows a low frequency of occurrence of this species in the natural environment (Begossi, et al. 1999; Pains da Silva et al. 2010; Horn et al. 2011; Súarez et al. 2013; Silveira and Weiss 2014). Considering that most of the specimens are caught in the wild for trade rather than captive breeding, a condition of overexploitation of specimens may lead to population decline (Cooney et al. 2015).

DNA was extracted from the muscle tissue of a specimen *A. hypselonotus,* acquired from an aquarium (S −19.9439W −43.9325), in the city of Belo Horizonte, Minas Gerais, Brazil and deposited in the vertebrates collection of the Federal University of the West of the Bahia (Voucher UFOB:VERT:0055), Bahia, Brazil. The genomic library was constructed and sequenced using MiSeq platform (Illumina, San Diego, CA.), with paired-end 150 bp strategy. The assembly was produced using Geneious Prime 2019.0.4.

The complete mitochondrial genome of *A. hypselonotus* (accession Genbank MW541938) was 16,685 bp in length. The base composition is estimated 29,69% for A, 28,17% for T, 26,41% for C and 15,75% for G, and the result of GC content was 42,14%. The mitochondrial genome was annotated using MitoFish webserver (Iwasaki et al. 2013) and Expasy Translation tool (https://web.expasy.org/translate/) (Artimo et al. 2012) was used for verification of the protein sequence. The mtDNA contain 13 protein-coding genes (PCGs), 2 rRNAs genes, 22 tRNAs genes and one control region.

The gene arrangement and coding bands were similar to the vertebrate standard mitogenome (Satoh et al. 2016). It has two PCGs with GTG start codon (*CoxI* and *Nd4*) and the others with ATG start codon. Four of the 13 PCGs contain TAA stop codon (*Nd1, ATP8, Nd4L* and *Nd6*), three contain incomplete TA_ stop codon (*Nd2, ATP6* and *CoxIII*), four contain incomplete T_ _ stop codon (*CoxII, Nd3, Nd4* and *Cytb*), one contain AGG stop codon (*CoxI*) and one TAG stop codon (*Nd5*). The incomplete stop codons were completed as TAA by post-transcriptional polyadenylation (Ojala et al. 1981). Intergenic regions ranged from 1 to 14 bp. Base overlap was found between the tRNA^IIe^ and tRNA^Gln^ (2 bp); tRNA^Gln^ and tRNA^Met^ (1 bp); *CoxI* gene and tRNA^Ser^ (13 bp); *ATP8* and *ATP6* genes (10 bp); *Nd4L* and *Nd4* genes (10 bp); *Nd5* and *Nd6* genes (4 bp); tRNA^Thr^ and tRNA^Pro^ (2 bp). Size of the tRNAs ranged from 66 to 74 bp and control region appeared 1,028 bp in length.

The phylogenetic tree was constructed based on the comparison of the 13 mitochondrial PCGs sequences of *Abramites hypselonotus* and other fish species of Characiformes (9 species) and Siluriformes (4 species - outgroup) orders, using the Maximum Likelihood method, with 1000 bootstrap replicates, on MEGA 6 Program. *Abramites hypselonotus* grouped with *Megaleporinus piavussu* (NC_025767.1), *Megaleporinus obtusidens* (NC_034945.1), *Megaleporinus elongatus* (NC_034281.1), and together they formed a sister group of *Leporinus affinis* (AP011994.1), thus maintaining the Family Anostomidae as a clade (Figure 1).

**Figure 1.**
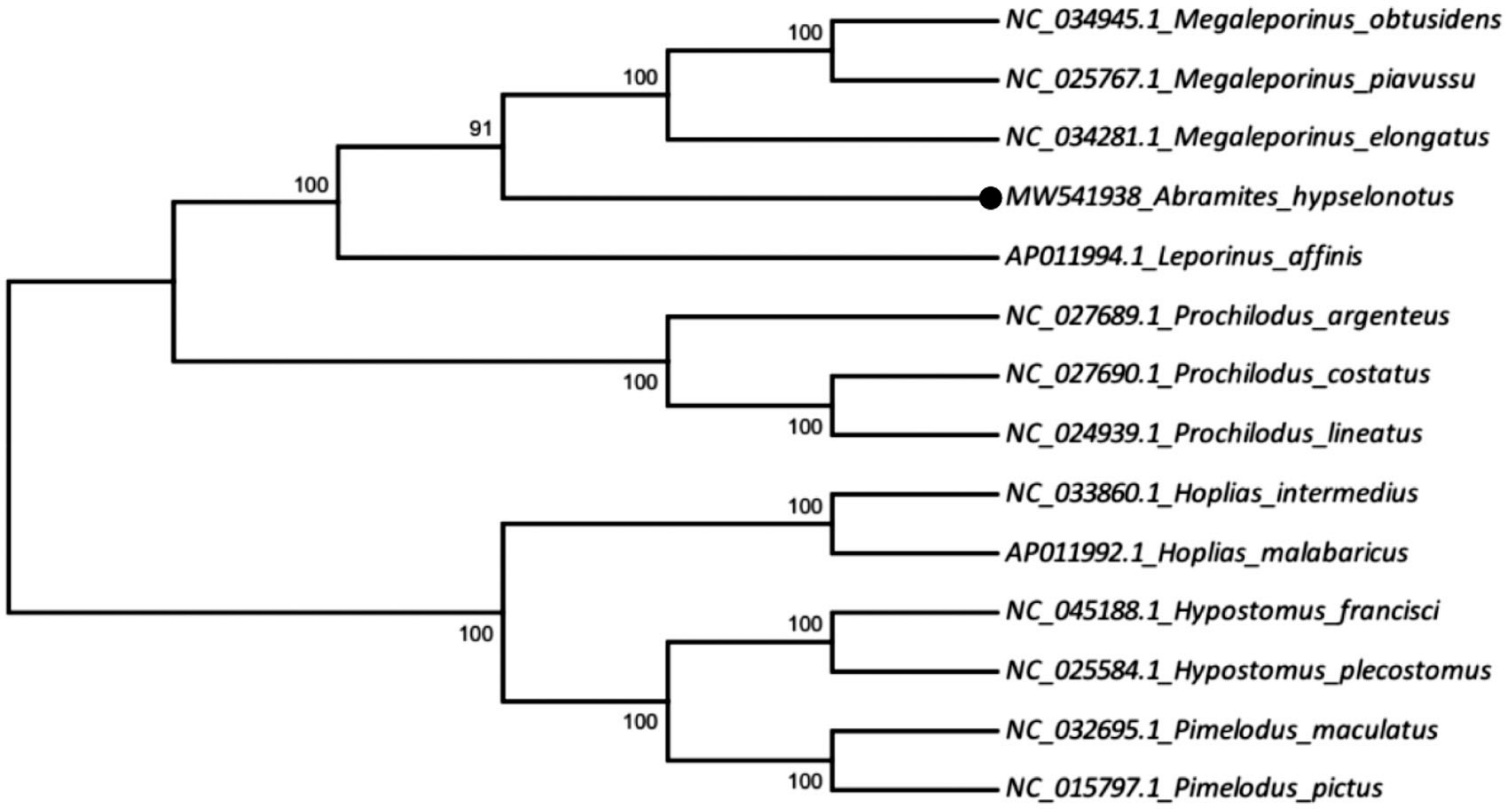
The phylogenetic tree was constructed based on the maximum likelihood method (ML) using 13 mitochondrial PCGs and shows the position of *Abramites hypselonotus* (MW541938) in Characiformes order and in Anostomidae family. Four species of the Siluriformes order (NC_045188.1 *Hypostomus francisci*, NC_025584.1 *Hypostomus plecostomus*, NC_032695.1 *Pimelodus maculatus*, NC_015797.1 *Pimelodus pictus*) were included as outgroup.

The mitogenome of *Abramites hypselonotus*, with 16,685 bp, is similar to other characiform fish, but it presents a difference in the number overlapping bases between the *Nd4L-Nd4* genes (10 bp).

## Data Availability

The genome sequence data that support the findings of this study are openly available in GenBank of NCBI at https://www.ncbi.nlm.nih.gov under the accession no. MW541938. The associated BioProject, SRA, and Bio-Sample numbers are PRJNA698912, SRR13614799, and SAMN17762312 respectively.
